# Evolved pesticide tolerance influences susceptibility to parasites in amphibians

**DOI:** 10.1111/eva.12500

**Published:** 2017-07-04

**Authors:** Jessica Hua, Vanessa P. Wuerthner, Devin K. Jones, Brian Mattes, Rickey D. Cothran, Rick A. Relyea, Jason T. Hoverman

**Affiliations:** ^1^ Biological Sciences Department Binghamton University (SUNY) Binghamton NY USA; ^2^ Department of Biological Sciences Rensselaer Polytechnic Institute Troy NY USA; ^3^ Biological Sciences Department Southwestern Oklahoma State University Weatherford OK USA; ^4^ Department of Forestry and Natural Resources Purdue University West Lafayette IN USA

**Keywords:** acetylcholineesterase inhibitor, carbaryl, ecotoxicology, *Lithobates sylvaticus*, pesticide–parasite interactions

## Abstract

Because ecosystems throughout the globe are contaminated with pesticides, there is a need to understand how natural populations cope with pesticides and the implications for ecological interactions. From an evolutionary perspective, there is evidence that pesticide tolerance can be achieved via two mechanisms: selection for constitutive tolerance over multiple generations or by inducing tolerance within a single generation via phenotypic plasticity. While both mechanisms can allow organisms to persist in contaminated environments, they might result in different performance trade‐offs including population susceptibility to parasites. We have identified 15 wood frog populations that exist along a gradient from close to agriculture and high, constitutive pesticide tolerance to far from agriculture and inducible pesticide tolerance. Using these populations, we investigated the relationship between evolutionary responses to the common insecticide carbaryl and host susceptibility to the trematode *Echinoparyphium* lineage 3 and ranavirus using laboratory exposure assays. For *Echinoparyphium*, we discovered that wood frog populations living closer to agriculture with high, constitutive tolerance experienced lower loads than populations living far from agriculture with inducible pesticide tolerance. For ranavirus, we found no relationship between the mechanism of evolved pesticide tolerance and survival, but populations living closer to agriculture with high, constitutive tolerance experienced higher viral loads than populations far from agriculture with inducible tolerance. Land use and mechanisms of evolved pesticide tolerance were associated with susceptibility to parasites, but the direction of the relationship is dependent on the type of parasite, underscoring the complexity between land use and disease outcomes. Collectively, our results demonstrate that evolved pesticide tolerance can indirectly influence host–parasite interactions and underscores the importance of including evolutionary processes in ecotoxicological studies.

## 1 INTRODUCTION

For over 70 years, pesticide use has improved human health and enhanced agricultural yields by reducing populations of disease vectors and pest species (Pimentel, 2005). One major consequence of pesticide use is the evolution of pesticide tolerance in pest species (Georghiou, 1990; Hoy, 1998; Weill et al., 2003). For nearly every major class of agrochemical, the evolution of tolerance has been reported in at least one pest species (Pimentel, 2005). Additionally, the evolution of pesticide tolerance in pest species is estimated to cause >$1.5 billion in crop losses each year (Pimentel, 2005). Despite our increasing knowledge of evolved pesticide tolerance in pest species, few studies have explored whether pesticide tolerance has evolved in nontarget species that are inadvertently exposed to these chemicals (Brausch & Smith, 2009a,b; Jansen, Coors, Stoks, & De Meester, 2011). Pesticide application for pest control is typically localized; however, processes such as aerial drift, runoff, and movement through food webs can distribute pesticides across the landscape potentially shaping the evolutionary courses of natural populations (Gilliom, 2007; Stone, Gilliom, & Ryberg, 2014). The potential for nontarget organisms to evolve tolerance is critical to consider in toxicological assays because it affects estimated toxicity metrics and therefore risk assessments conducted for nontarget species.

There is increasing evidence that nontarget populations can evolve increased tolerance to pesticides (Bendis & Relyea, 2014; Brausch & Smith, 2009a; Cothran, Brown, & Relyea, 2013; Hua et al., 2015; Jansen et al., 2011). Theory suggests that the evolution of pesticide tolerance can be achieved via two mechanisms: selection for constitutive tolerance over multiple generations or by inducing tolerance within a single generation via phenotypic plasticity (Cothran et al., 2013; Hua et al., 2015; Jansen et al., 2011). The evolution of constitutive tolerance is more likely when populations are in close proximity to agriculture (<200 m) and consistently exposed to pesticides (Crispo, 2007; Declerck et al., 2006). In contrast, inducible tolerance is more likely when populations are far from agriculture, infrequently exposed to pesticides, and there are costs of maintaining pesticide tolerance when pesticides are not present (i.e., phenotypic trade‐off; Crispo, 2007; Crispo et al., 2010). While constitutive and inducible pesticide tolerance can improve survival following pesticide exposure, both mechanisms can alter how organisms interact with other community members (i.e., competition, predator–prey, host–parasite interactions; Gotthard & Nylin, 1995; Hanazato, 1995; McCarroll & Hemingway, 2002; Raberg, 2014). Thus, to advance the field of toxicology, there is a need to not only consider how evolutionary responses to pesticides affect estimates of pesticide toxicity, but also how these evolutionary responses alter ecological interactions.

The influence of pesticides on host–parasite interactions has been explored in many systems (Blaustein et al., 2011; Dietrich, Van Gaest, Strickland, & Arkoosh, 2014; Doublet, Labarussias, de Miranda, Moritz, & Paxton, 2015). Several studies suggest that the interaction between pesticides and parasites can contribute to declines in wild populations either directly (e.g., mortality) or indirectly when the presence of the pesticide alters the traits of the parasite or the host (e.g., immunosuppression; Christin et al., 2003; Kiesecker, 2002; Rohr, Raffel, Sessions, & Hudson, 2008). Fundamentally different from these mechanisms is the possibility that populations with evolved constitutive or inducible tolerance to pesticides experience altered host–parasite interactions, even in the absence of the pesticide, due to phenotypic trade‐offs.

Theory predicts that populations that express constitutive or inducible pesticide tolerance may face nonadaptive trade‐offs (i.e., increased susceptibility to parasites) or beneficial correlated traits that improve host resistance and tolerance to parasites (Auld, Agrawal, & Relyea, 2010; Dewitt, Sih, & Wilson, 1998; Gotthard & Nylin, 1995; Hanazato, 1995; McCarroll & Hemingway, 2002; Raberg, 2014). For example, the evolution of constitutive resistance to pyrethroid insecticides in mosquitoes (*Anopheles gambiae*) is associated with the production of a thicker cuticle that shields individuals from chemical exposure (Balabanidou et al., 2016) and potentially from parasites that infect hosts via cuticle penetration (i.e., fungi, *Beauveria bassiana;* Yassine, Kamareddine, & Osta, 2012). In contrast, *A. gambiae* that achieve tolerance via target‐site insensitivity (i.e., knock‐down resistance; Protopopoff et al., 2013) are more susceptible to *Plasmodium* (Alout et al., 2016; Ndiath et al., 2014). These studies demonstrate that the different mechanisms of pesticide tolerance can differentially shape disease outcomes in target species. However, no studies have explored these questions in nontarget organisms.

### 1.1 Previous studies of inducible versus constitutive pesticide tolerance in amphibians

**Figure 1:**
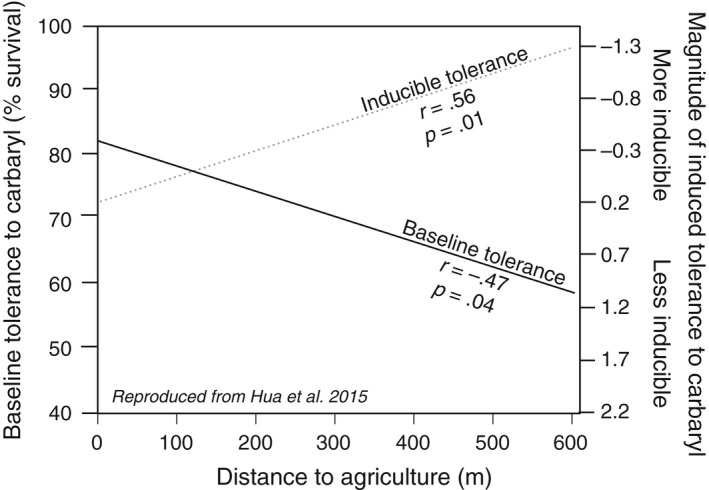
The relationship between distance to agriculture, magnitude of baseline tolerance, and magnitude of inducible tolerance to carbaryl. Regression lines are reproduced using data from Hua et al. (2015). Distance to agriculture (m) was calculated as the shortest distance from collection location to an agricultural field. The measure of baseline tolerance was calculated as the percent survival following exposure to a lethal concentration of carbaryl. The measure of inducible tolerance is represented by the hazard regression coefficient (more negative values indicate populations with higher plasticity to pesticides). Exact values are reported Table 1

Over the last several years, we have used wood frogs (*Lithobates sylvaticus*) to explore patterns in pesticide tolerance (Hua, Cothran, Stoler, & Relyea, 2013; Hua, Jones, & Relyea, 2014; Hua et al., 2015). Wood frog populations inhabit pond ecosystems that can encounter pesticides following direct application, runoff, or aerial drift (Smalling et al., 2015). Using 15 populations in northwestern Pennsylvania (Hua et al., 2013, 2014, 2015), we found that populations living close to agriculture have higher baseline tolerance to a common acetylcholineesterase (AChE) inhibiting pesticide (carbaryl) compared to populations living far from agriculture (Hua et al., 2015). Using the same populations, we also investigated patterns of plasticity to carbaryl and found that the degree of plasticity to carbaryl varied along an agricultural gradient; populations close to agriculture possess high baseline but no inducible tolerance to carbaryl (i.e., constitutive tolerance), whereas populations far from agriculture possess low baseline but high inducible tolerance to carbaryl (Figure 1). Additionally, we have demonstrated that these landscape patterns were generalizable; wood frog populations with constitutive and inducible tolerance to carbaryl also had constitutive and inducible cross‐tolerance to other AChE inhibitors (Hua et al., 2013, 2014).

Collectively, the spatial patterns of constitutive and inducible tolerance provide a useful framework for exploring the relationship between the mechanisms of pesticide tolerance and parasite susceptibility. Using experimental parasite exposures, here we hypothesized that in an environment without pesticides, populations with constitutive tolerance (i.e., populations close to agriculture with high baseline tolerance to pesticides) should be more susceptible to both parasite species compared to populations with inducible tolerance (i.e., living far from agriculture with low baseline tolerance).

## 2 METHODS

### 2.1 Model parasites

We chose to work with echinostomes and ranavirus, two common amphibian parasites (Hoverman, Mihaljevic, Richgels, Kerby, & Johnson, 2012). Echinostomes (e.g., *Echinostoma trivolvis, Echinoparyphium* sp.) are widespread trematodes that use larval amphibians as secondary intermediate hosts (Huffman & Fried, 2012; Schell, 1985). In larval amphibians, echinostomes encyst in the kidneys (Smyth & Halton, 1983). Consistent with other macroparasites, the effect of echinostomes on hosts is dose‐dependent (e.g., hemorrhaging, edema, mortality; Huffman & Fried, 2012). Amphibian species vary in their susceptibility to echinostomes (Sears, Schlunk, & Rohr, 2012), and past work suggests that tadpole species with shorter larval periods, such as wood frogs, are more susceptible to infection by echinostomes (Johnson et al., 2012; Rohr, Raffel, & Hall, 2010).

Ranaviruses are emerging viral pathogens that can infect fish, amphibians, and reptiles. In amphibians, ranavirus is widely distributed and has been implicated in mortality events across the globe (Chinchar, Hyatt, Miyazaki, & Williams, 2009; Duffus et al., 2015). Larvae and recently metamorphosed individuals are more frequently reported in mortality events than adults (Brunner, Storfer, Gray, & Hoverman, 2015; Duffus et al., 2015). Ranavirus can lead to host mortality by initiating cell death within the liver, kidney, and spleen (Gray, Miller, & Hoverman, 2009; Jancovich, Qin, Zhang, & Chinchar, 2015). Wood frogs are one of the most susceptible amphibian species to ranavirus (Hoverman, Gray, Haislip, & Miller, 2011).

### 2.2 Animal collection and husbandry

We collected wood frog eggs from 15 populations (Table 1) in northwestern Pennsylvania. The populations were separated by at least 4 km as past studies demonstrate that the genetic neighborhood is generally within ~1 km of the breeding pond (Berven & Grudzien, 1990; Semlitsch, 2000; Semlitsch & Bodie, 1998). On April 8, 2014, we collected 10 egg clutches (Gosner stage 3–7; Gosner, 1960) from each population. On 21 April, the eggs (Gosner stage 18) were shipped overnight to the Purdue Wildlife Area (PWA) in West Lafayette, IN. We placed all masses into in 100‐L pools filled with 90 L of well water, keeping eggs from the same populations together. By 28 April, all tadpoles reached Gosner stage 25 and were fed rabbit chow ad libitum until the start of the experiments.

**Table 1:** The abbreviations, coordinates, distance to agriculture (m), measure of constitutive tolerance (percent survival following exposure to carbaryl), and measure of inducible tolerance (hazard regression coefficient; the more negative values indicate populations with higher pesticide‐induced tolerance) for the wood frog populations used in this study. All values are derived from Hua et al. (2015)

Pond ID	Latitude (N)	Longitude (W)	Distance to agriculture	Constitutive tolerance	Inducible tolerance
BJ	41°39.9′	80°30.8′	300	60	−0.67
BOR	41°55.2′	80°1.9′	315	84	−0.72
BOW	41°55.6′	79°48.2′	452	80	−0.95
GRV	41°41.0′	80°2.8′	201	72	−0.50
HOP	41°52.1′	80°28.0′	559	52	−1.20
LOG	41°58.1′	79°36.1′	12.6	58	−0.81
RR	41°36.4′	80°22.9′	436	60	−0.97
REE	41°58.9′	79°58.2′	256	64	−0.59
ROA	41°53.1′	79°36.3′	183	88	0.53
SKN	41°59.9′	79°46.5′	11.5	72	−0.52
SQR	41°50.5′	80°14.4′	412	76	−0.94
STB	41°35.4′	80°25.9′	55	96	2.04
TRL	41°34.1′	80°27.1′	65.6	80	−0.50
TT	41°37.8′	79°54.7′	430	76	−0.52
XTI	41°37.6′	80°27.7′	600	44	−1.33

### 2.3 Trematode experiment

To obtain *Echinoparyphium* for the experiments, we collected adult ramshorn snails (*Helisoma trivolvis*), the first intermediate host of the trematode, from the PWA. We screened the snails for *Echinoparyphium* infection by isolating individuals in 50‐ml tubes filled with 45 ml of aged, UV‐irradiated well water and placed the tube under a light source for one hour to induce the shedding of the free‐swimming cercariae (Hua, Buss, Kim, Orlofske, & Hoverman, 2016). We selected five snails that shed the highest density of cercariae and housed them separately in 2‐L plastic containers with 1.5 L of aged, UV‐irradiated well water at 7°C to slow shedding of cercariae until the start of the experiment. Three days prior to the start of the experiment, snails were acclimated to 25°C and fed rabbit chow ad libitum. We identified cercariae as *Echinoparyphium* lineage 3 using standard molecular sequencing of the ITS1 gene and implementation of Bayesian phylogenetics methods (Detwiler, Bos, & Minchella, 2010; Hua et al., 2016).

To investigate the susceptibility of tadpoles to trematodes, we selected 20 tadpoles of a similar size from each population and allowed them to acclimate to indoor conditions for 24 hr on 18 May. During this acclimation period, we haphazardly distributed tadpoles into 1‐L plastic containers filled with 500 ml of aged, UV‐irradiated well water (pH 7.4; 19.1°C; 14:10 light:dark cycle). Each container contained a single tadpole, and we fed all tadpoles TetraMin (Tetra Spectrum Brands) ad libitum. On 19 May, we individually exposed 15 tadpoles from each of the 15 populations to 50 free‐swimming trematodes (cercariae) for a total of 225 experimental units. To account for any potential background mortality, we also included a no‐trematode control for each population, which was replicated five times for an additional 75 experimental units.

To obtain the cercariae, we placed the previously collected infected snails into 50‐ml tubes filled with 45 ml of aged, UV‐irradiated well water under a light source to induce shedding. We then used a stereo dissecting scope and glass pipette to isolate 50 cercariae into individual 50‐ml Falcon tubes containing 45 ml of aged, UV‐irradiated aged well water. Within 4 hr of counting, we added the cercariae to each experimental unit. To ensure that all cercariae were added to each treatment, each tube was rinsed three times using water from the experimental unit. For the control treatment, we repeated this procedure by adding the same volume of water from an uninfected snail.

Tadpoles were checked daily for mortality and fed TetraMin ad libitum. We terminated the experiment 3 days postexposure because past studies demonstrate this is enough time for parasites to successfully encyst and before parasite clearance begins (Hoverman, Hoye, & Johnson, 2013). Tadpoles were euthanized using a MS‐222 overdose and then preserved in 10% formalin. We weighed, staged, and measured snout‐vent length for each individual (Table S1). The tadpoles were then dissected to quantify parasite load. We then calculated the proportion of trematodes encysted (i.e., number of encysted trematodes out of the 50 cercariae added to each experimental unit). We scanned the entire body of the tadpole for encysted *Echinoparyphium* but found all metacercariae in the kidneys. We also examined all control individuals to confirm the absence of trematode infection; all individuals were negative.

### 2.4 Ranavirus experiment

We used a FV3‐like isolate in our experiment that was obtained from a die‐off of larval salamanders in the Great Smoky Mountains National Park, TN. This isolate was passaged through fathead minnow cells fed with Eagle's minimum essential medium (MEM) with Hank's salts, containing 5% fetal calf serum. The virus was on the third passage since original isolation and was stored at −80°C until the start of the experiment.

We examined the effects of ranaviruses using a factorial experiment that crossed 14 wood frog populations with the presence or absence of ranavirus exposure (0 or 10^3^ plaque forming units [PFUs]/ml). Each of the 28 treatments was replicated four times for a total of 112 experimental units. Experimental units were 2‐L plastic containers filled with 1 L of aged, UV‐irradiated well water with 10 tadpoles per unit. We conducted the experiment using 14 populations because there were not enough animals available from the STB population.

On 28 May, we selected 80 tadpoles of a similar size from each of the 14 populations and allowed them to acclimate to indoor conditions for 24 hr. During this acclimation period, we haphazardly distributed the tadpoles into groups of 10 and held them in 2‐L plastic containers filled with 1 L of aged, UV‐irradiated well water (pH 7.4; 19.1°C; 14:10 light:dark cycle) and fed TetraMin ad libitum. On 29 May, keeping tadpoles in the same units (10 tadpoles/unit), we added 625 μl of the virus (original titer 1.6 × 106 PFUs/ml) to each experimental unit assigned the virus treatment to achieve a final concentration of 10^3^ PFUs/ml. For the control treatment, we added 625 μl of MEM to the experimental units. Tadpoles were monitored every 12 hr for mortality, fed TetraMin ad libitum every 2 days, and water was changed every 5 days without virus renewal to maintain water quality. Eleven days after the addition of the virus, we assessed final survival, euthanized all surviving tadpoles using a MS‐222 overdose and preserved tadpoles in 70% EtOH to quantify viral load. We chose 11 days for the ranavirus exposure because past work has shown that this time period is sufficient to observe disease outcomes (infection and mortality) in wood frog tadpoles (Hoverman et al., 2011).

Prior to quantifying viral loads, we weighed, staged, and measured snout‐vent length for each individual (Table S2). Then, we dissected each individual to remove the kidneys and liver to quantify viral load. For each individual, the tissues were combined into a 1.5‐ml microcentrifuge tube and stored at −80°C for qPCR analysis and DNA quantification. In addition, we examined 10% of all control individuals to confirm the absence of ranavirus infection; all individuals were negative. To prevent cross‐contamination, all tools and surfaces were soaked in 10% bleach for 10 min and gloves were changed between each dissection.

To assess infection status on each individual, we first conducted DNA extractions using DNeasy Blood and Tissue Kits (Qiagen). Then, we used quantitative polymerase chain reaction (qPCR) to determine virus infection status and viral load of each individual following the methods of Forson and Storfer (2006). The qPCR mixture included 6.25 μl of TaqMan^®^ Universal PCR Master Mix (Applied Biosystems), 1.0 μl of a mixture of each primer at 10 pmol/μl (rtMCP‐F [5′‐ACA CCA CCG CCC AAA AGT AC‐3′] and rtMCP‐R [5′‐CCG TTC ATG ATG CGG ATA ATG‐3′]) and a fluorescent probe rtMCP‐probe (5′‐ CCT CAT CGT TCT GGC CAT CAA CCA‐3′). We added 2.5 μl of template DNA and DNA grade water to a final volume of 12.25 μl. The qPCR was performed using a Bio‐Rad real‐time qPCR system (Bio‐Rad). In each qPCR run, we included a standard curve and a negative virus‐free water sample. We used a synthetic double‐stranded DNA standard by synthesizing a 250‐bp fragment of the major capsid protein (MCP) gene (gBlocks Gene Fragments; Integrated DNA Technologies), which is conserved among *Ranavirus* species. We prepared a log‐based dilution series (4.014 × 10^9^–4.014 × 10^6^ viral copies/μl) for the standard curve. Standard curve samples and unknowns were run in duplicate. All duplicated unknowns that peaked before cycle 40 were considered positive. Following qPCR, we quantified the concentration of genomic DNA in the samples (ng of DNA/μl) using a NanoDrop 2000c (Thermo Scientific). We then used the genomic DNA concentration along with the viral concentration data to calculate the viral load (i.e., viral copies/ng of DNA) of the positive samples.

### 2.5 Statistical analysis

Distance to agriculture, baseline pesticide tolerance, and the magnitude of inducible tolerance are highly correlated factors that contribute to evolved pesticide tolerance in natural wood frog populations (Hua et al., 2015). Therefore, we conducted a factor reduction analysis using Principal Axis Factoring with an orthogonal Varimax rotation (SPSS; factor reduction) to facilitate interpretations of the relationship between the mechanism of pesticide tolerance and susceptibility to parasites. Using the Guttman–Kaiser criterion (i.e., the number of factors are determined by those with eigenvalues over 1.0; Yeomans & Golder, 1982), we reduced our three variables into a single predictor (PC‐1). Values for distance to agriculture, baseline pesticide tolerance, and inducible tolerance were obtained from a previous study that tested wood frogs from the same egg masses used in this study (Hua et al., 2015; Table 1). Due to differences in the total number of populations tested in the trematode and ranavirus experiment (15 vs. 14, respectively), we conducted two separate factor reduction analysis and calculated two separate PC‐1 for the trematode and ranavirus experiments.

For the trematode experiment, we conducted a regression analysis (SPSS Regression) to assess the relationship between PC‐1 for the 15 populations and trematode resistance (average proportion of trematodes encysted). We did not conduct a regression analysis assessing the relationship between PC‐1 and tadpole survival because all tadpoles survived the trematode and no‐trematode exposures.

For the ranavirus experiment, we conducted four regression analyses (SPSS regression). The first analysis assessed the relationship between PC‐1 for the 14 populations and average percent tadpole survival following a 120‐hr exposure to ranavirus. The second analysis assessed the relationship between PC‐1 and average tadpole time to death (TTD). The third analysis assessed the relationship between percent survival and viral load, using a binomial logistic regression (SPSS Logistic regression). Because an individual's survival is directly related to its viral load, we conducted our logistic regression on individual tadpoles rather than population means. To account for the potential effect of experimental units, we included experimental unit as a covariate. Finally, we conducted a fourth regression analysis to determine the relationship between PC‐1 and ranavirus load. For this analysis, we conducted two separate linear regressions to account for differences in the viral load of individuals that survived versus those that did not survive. For both linear regressions, we conducted our analyses on the average load of tadpoles from each population. We accidentally did not add ranavirus to one of the replicates from the SKN population, so we removed this replicate from the analysis. All variables in the linear regression analyses met assumptions of normality (Shapiro‐Wilk; *p* > .05).

## 3 RESULTS

### 3.1 Factor reduction analysis

For the trematode and ranavirus experiments, Bartlett's test of sphericity confirmed a patterned relationship between variables (*p *< .003 and *p* < .009, respectively). Kaiser–Meyer–Olkin (KMO) measure of sampling adequacy for the trematode and ranavirus experiments were above the 0.5 cutoff point (0.66 and 0.61, respectively). Populations with more negative factor scores were those that lived farther from agriculture, had lower baseline tolerance, and had higher inducible tolerance. In contrast, populations with more positive factor scores were those that lived closer to agriculture, had higher baseline tolerance, but had lower inducible tolerance. Factor loadings, eigenvalues, percent variance explained, and factor scores for both experiments are reported in Tables S3 and S4).

### 3.2 Trematode experiment

**Figure 2:**
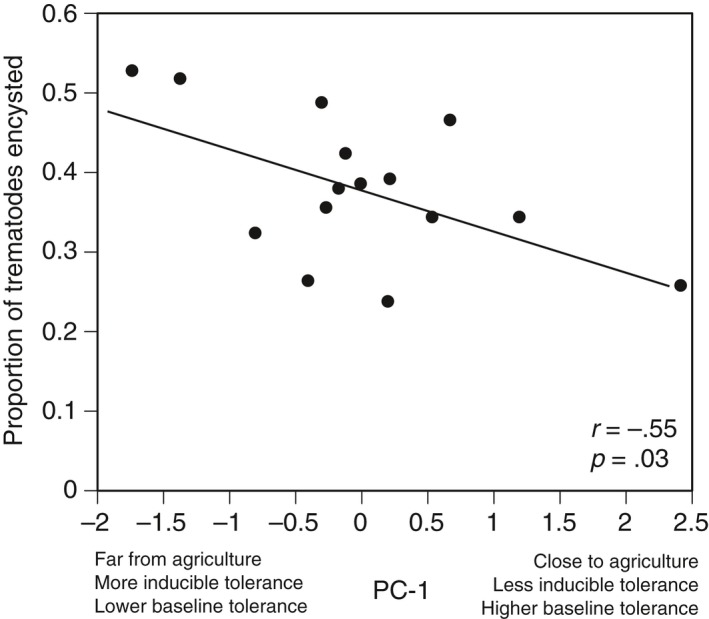
The relationship between PC‐1 and the average proportion of trematodes encysted in wood frog tadpoles from 15 populations (*r* represents the correlation coefficient). To create PC‐1, we reduced our three variables (distance to agriculture, magnitude of baseline tolerance, and magnitude of inducible tolerance) into a single predictor (PC‐1). Each point represents the average proportion of trematodes that successfully encysted in tadpoles from a population

We had 100% survival in the no‐trematode and trematode exposure treatments. The proportion of trematodes encysted varied across the 15 populations with population averages ranging from 23.8% to 52.8% (Figure 2; trematode loads reported in Fig. S1). We found a significant negative relationship between PC‐1 and the proportion of trematodes encysted in tadpoles from each population (*r *=* *−.55; *p *=* *.03; Figure 2). Populations that live closer to agriculture and have higher baseline tolerance had a lower proportion of trematodes encysted compared to tadpoles that live further from agriculture and have higher inducible tolerance.

### 3.3 Ranavirus experiment

**Figure 3:**
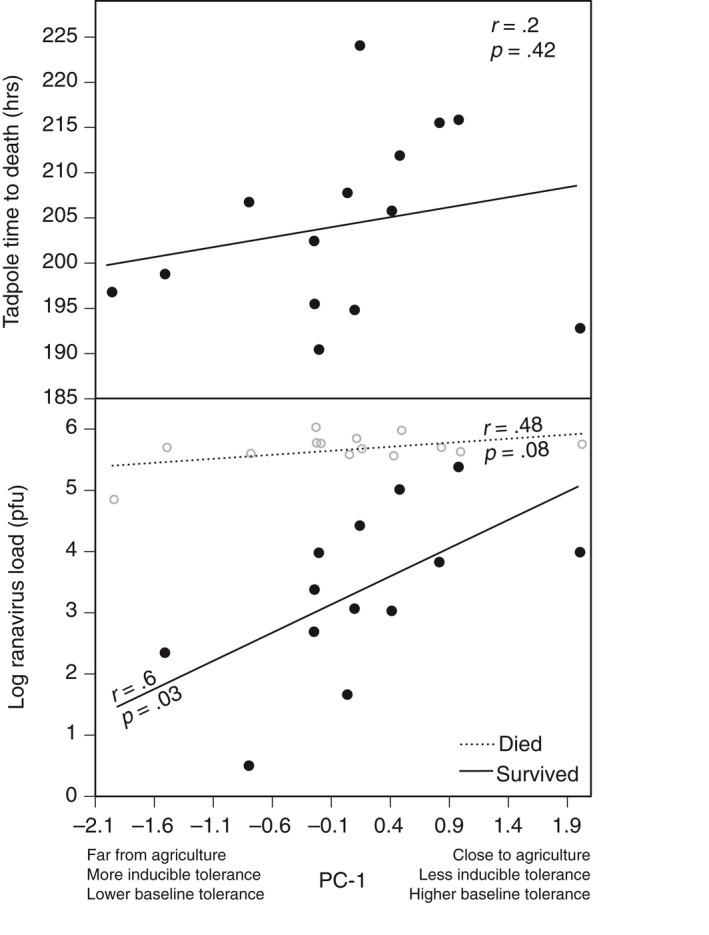
The relationship between PC‐1 and average tadpole time to death (top panel) and PC‐1 and ranavirus load (log‐transformed; bottom panel) for infected wood frog tadpoles that survived (solid line) and died (dotted line) from 14 populations (*r* represents the correlation coefficient). To create PC‐1, we reduced our three variables (distance to agriculture, magnitude of baseline tolerance, and magnitude of inducible tolerance) into a single predictor (PC‐1). Each point represents the average log‐transformed viral load of all individuals from a population

Tadpoles not exposed to ranavirus experienced 99.6% survival. Therefore, we did not examine the relationship between survival of tadpoles not exposed to ranavirus and PC‐1. For tadpoles exposed to ranavirus, average survival, time to death, and log viral load of each population ranged from 0% to 4.7%, 190–224* *hr, and 4.6–5.9 viral copies/ng of DNA, respectively (Fig. S2). We found no relationship between PC‐1 and mean tadpole survival among populations exposed to ranavirus (*r *= .11; *p *= .70; Fig. S3) nor between PC‐1 and mean tadpole TTD among the populations exposed to ranavirus (*r *= .20; *p *= .42; Figure 3). However, survival was related to ranavirus load (χ^2^ = 249.8; *p* < .001; *df* = 2; Fig. S4); tadpoles with higher ranavirus loads were less likely to survive than tadpoles with lower loads (*p* < .001). The logistic regression model explained 61.5% (Nagelkerke R) of the variance in survival and correctly classified 92.8% of cases. There was no significant effect of experimental unit (*p* = .328).

Finally, for tadpoles that survived ranavirus exposure, we found a significant positive relationship between PC‐1 and ranavirus load (*r* = .60; *p* = .03; Figure 3). Populations that live closer to agriculture and have higher baseline tolerance had higher viral loads compared to tadpoles that live further from agriculture and have higher inducible tolerance. This relationship was not influenced by differences in the number of individuals surviving from inducible versus constitutive populations (Fig. S3). For tadpoles that died following ranavirus exposure, we found a marginally significant, positive relationship between PC‐1 and ranavirus load (*r* = .48; *p* = .08; Figure 3).

## 4 DISCUSSION

Using 15 wood frog populations, we demonstrated that differences in evolved pesticide tolerance associated with proximity to agriculture influenced susceptibility to parasites, but the patterns depended on the type of parasite. When exposed to trematodes, populations closer to agriculture with less inducible tolerance and higher baseline tolerance were less susceptible to trematodes (as indicated by a lower proportion of trematodes encysted) compared to populations that are further from agriculture with more inducible tolerance and lower baseline tolerance. In contrast, for individuals that survived the ranavirus experiment, those from populations closer to agriculture with lower inducible tolerance and higher baseline tolerance had higher viral loads compared to those populations further from agriculture with more inducible tolerance and lower baseline tolerance.

Populations closer to agriculture with less inducible tolerance and higher baseline tolerance were more resistant to *Echinoparyphium* (lower proportion of trematodes encysted) compared to populations further from agriculture with more inducible tolerance and lower baseline tolerance. While the mechanisms underlying this relationship are unknown, research on mosquitoes has found that constitutive pesticide tolerance was associated with an alteration of the redox potential within mosquito tissue, which ultimately created a toxic environment for embedded roundworm larvae (McCarroll & Hemingway, 2002). In a study using the toad *Rhinella arenarum*, researchers found that exposure to malathion, an AChE inhibitor, also caused a modification in cell redox potential (Anguiano, Caballero de Castro, & Pechen de D'Angelo, 2001). Thus, one hypothesis for our results is that selection for constitutive tolerance—but not inducible tolerance—indirectly influences trematode resistance by initiating an internal environment that is toxic to trematodes. Given that host pathology (e.g., hemorrhaging, edema, mortality) is dose‐dependent for *Echinoparyphium*, a reduction in trematode load could increase host fitness (Huffman & Fried, 2012; Orlofske, Belden, & Hopkins, 2009). However, our study focused on short‐term resistance to trematode infection. While individuals from populations further from agriculture with higher inducible tolerance and lower baseline tolerance were less resistant to trematodes, they could display higher levels of parasite clearance or tolerance compared to individuals from populations closer to agriculture with lower inducible tolerance and higher baseline tolerance (Raberg, 2014). Integrating short‐term (i.e., resistance) and long‐term parameters (i.e., clearance and tolerance) will provide a more complete understanding of how evolved pesticide tolerance influences host–trematode interactions.

We found that 100% of the wood frog tadpoles that were exposed to ranavirus became infected. Average final survival varied minimally across the populations (0% to 4.7%) suggesting that all populations, regardless of pesticide tolerance mechanism, were highly susceptible to ranavirus. This is consistent with past work demonstrating that wood frogs are one of the most susceptible amphibian species to ranavirus infection (Hoverman et al., 2011). Given the limited variability in survival across the populations, it is not surprising that we did not find a relationship with the mechanism of pesticide tolerance. However, we also measured time to death (TTD), which provides an alternative metric for measuring susceptibility to ranavirus. Although average TTD across the 14 populations varied more than survival (TTD ranging from 190 to 224 hr), we still did not find a relationship between the mechanism of pesticide tolerance and susceptibility to ranavirus. Amphibian species with intermediate levels of susceptibility to ranavirus (e.g., hylids; Hoverman et al., 2011) may be better suited to address our hypotheses regarding the interaction with evolved pesticide tolerance because there is the potential for more variation in survival rates. However, wood frogs are the only amphibian species that has been examined for patterns in evolved pesticide tolerance.

While there was no relationship between the mechanism of pesticide tolerance and survival with ranavirus, we found a significant relationship between the mechanism of pesticide tolerance and ranavirus load. Ranaviral load is associated with increased mortality; thus, higher loads may result in decreased survival in the wild (Wuerthner, Hua, & Hoverman, 2017). For tadpoles that survived the ranavirus exposure, individuals from populations closer to agriculture with lower inducible tolerance and higher baseline tolerance had markedly higher viral loads than those further from agriculture with higher inducible tolerance and lower baseline tolerance. Indeed, we found a 230% increase in viral loads from the least to most susceptible population. In nature, wood frog tadpoles interact with a diversity of other amphibian species (Werner et al., 2014) and wood frogs are often implicated as important species in ranavirus transmission (Harp & Petranka, 2006). As such, the wide variation in viral loads between constitutive versus inducible populations may have broad implications for understanding transmission dynamics of ranavirus within communities.

We also found a significant positive relationship between tadpole mortality and viral load. Previous studies have reported a similar correlation between viral load and mortality (Wuerthner et al., 2017). Our results suggest that regardless of population, wood frogs experience mortality when log viral loads passed a threshold of approximately 5 viral copies/ng of DNA. A similar threshold was detected when log viral loads approached 4.5 viral copies/ng of DNA in gray tree frogs (*Hyla versicolor*; Wuerthner et al., 2017). Identifying a threshold value can potentially allow researchers to make predictions about the likelihood or timing of a ranavirus die‐off in natural populations.

Our results are consistent with the growing body of work demonstrating population‐level variation in the susceptibility of hosts to parasites (Bradley et al., 2015; Breitburg et al., 2015; Koprivnikar, Baker, & Forbes, 2006; Matson, 2006; Pearman & Garner, 2005; Schock, Bollinger, Chinchar, Jancovich, & Collins, 2008; Schock, Bollinger, & Collins, 2010). Further, this study provides the first evidence in a vertebrate species that evolutionary responses to pesticides influence susceptibility to parasites. Pesticide‐mediated effects on disease outcomes are commonly documented in the literature, yet the results are often variable with pesticides increasing host tolerance to parasite infection in some cases (Coors, Decaestecker, Jansen, & De Meester, 2008; Kiesecker, 2002; Rohr, Raffel, et al., 2008) but not in others (Griggs & Belden, 2008; Marcogliese et al., 2009; Schotthoefer et al., 2011). We demonstrate that the mechanism underlying the evolution of pesticide tolerance may be critical for assessing susceptibility to parasites. Moreover, we observed that the direction of the relationship varies depending on the parasite species. Thus, considering the evolutionary history of populations may shed light on the equivocal nature of pesticide–parasite interactions documented in the literature.

### 4.1 Future considerations

While we found that evolved pesticide tolerance can influence pesticide–parasite interactions, there are several important future research directions. For instance, our study focused on two parasite species and a single pesticide, host, and life stage. Because variation in these factors is known to influence disease outcomes (Bridges, 2000; Hammond, Jones, Stephens, & Relyea, 2012; Jones, Hammond, & Relyea, 2009; Kegley, Hill, Orme, & Choi, 2016), future work expanding upon this research is critical for identifying general trends and developing predictions about the role of evolved pesticide tolerance on host–parasite interactions.

We demonstrated a relationship between evolved pesticide tolerance and susceptibility to parasites, but the mechanisms driving these patterns are beyond the scope of this study. The consequences of evolutionary responses to pesticides on parasite susceptibility are more commonly addressed in studies of vector species. For example, in the mosquito, *A. gambiae*, the evolution of pesticide tolerance can arise via multiple mechanisms (e.g., production of a thicker cuticle or physiological defenses such as target‐site insensitivity; Balabanidou et al., 2016) that can lead to direct and indirect selection for traits that also confer resistance or tolerance to parasites. Therefore, interdisciplinary approaches that integrate discoveries in vector research may greatly facilitate our understanding of how evolutionary responses to pesticides influence susceptibility to parasites in nontarget species.

In this study, we focused only on assessing how variation in host tolerance to pesticides and proximity to agriculture affected disease outcomes. However, variation in the host, parasite, and environment (i.e., points in the disease triangle; Stevens, 1960; Budria & Candolin, 2014) all can influence disease outcomes. Similar to population‐level variation in the host populations, parasites can also vary in their tolerance to pesticides (Hua et al., 2016). Additionally, we conducted our study in pesticide‐free experimental environments, but if pesticides were present in the environment, the outcomes could be altered. Pesticides can perturb host–parasite interactions by directly reducing host or parasite abundance (i.e., density‐mediated effects). Pesticides in the environment may also alter traits, such as host immune responses, parasite infectivity, host behavior, or parasite behavior (i.e., trait‐mediated effects Rohr et al., 2003, 2004; Weis, Smith, Zhou, Santiago‐Bass, & Weis, 2001). Thus, future studies that consider evolutionary responses of parasites to pesticides and incorporate various environmental conditions would be valuable in determining the generalizability of the relationship we detected between evolved pesticide tolerance and parasite tolerance.

Our study focused on how evolved tolerance to pesticides affects host susceptibility to parasites. However, the reverse could be true as evolutionary responses to parasites could influence pesticide tolerance. For example, in mosquitoes, studies suggest that hosts that expended resources toward resisting infection by *Plasmodium* (i.e., detoxification enzymes) had increased vulnerability to insecticides (Saddler, Burda, & Koella, 2015). Past amphibian studies have also suggested scenarios that may lead to evolutionary responses to parasites. For example, high‐nutrient agricultural run‐off may indirectly facilitate trematode abundance by increasing the amount of algal resources available to the first intermediate hosts (snails) of trematodes (Koprivnikar & Redfern, 2012; Koprivnikar et al., 2006; Rohr, Schotthoefer, et al., 2008). Higher abundances of *Echinoparyphium* may select for increased resistance or tolerance to *Echinoparyphium* in amphibian populations near agriculture. Therefore, future studies that consider the possibility of evolved responses to parasites and their influence on disease outcomes would complement our understanding of how evolutionary processes affect responses to chemicals.

This study suggests that considering patterns of constitutive and inducible tolerance is critical in studies of toxicology as the predicted toxicity of a pesticide can depend on the evolutionary history of test populations. Evolutionary responses to pesticides not only effect estimates of pesticide toxicity but can also have indirect consequences by altering ecological interactions between hosts and their parasites. Therefore, considering evolutionary responses in toxicology is critical to understanding how contaminants affect ecosystems.

## DATA ARCHIVING STATEMENT

Data for this study are available at *Data available from the Dryad Digital Repository*: https://doi.org/10.5061/dryad.nb40v.
