# Supplementary material for: Evolved pesticide tolerance influences susceptibility to parasites in amphibians
Source: Evol Appl. 2017 Jul 4;10(8):802–12. doi: 10.1111/eva.12500 (PMC5680434; doi:10.1111/eva.12500)
Supplement: Supplementary file 1 [file EVA-10-802-s001.docx]

**Supplementary tables and figures**

**Tables**

Table S1: Mean tadpole traits (stage, mass, and snout-vent length (SVL)) ± SE across the 15 wood frog populations at the end of the 3-d trematode experiment.

| Population | Stage | Mass (mg) | SVL (mm) |
| --- | --- | --- | --- |
| BJ | 29.8 ± 0.1 | 177 ± 5 | 9.6 ± 0.1 |
| BOR | 28.4 ± 0.3 | 158 ± 8 | 9.5 ± 0.2 |
| BOW | 28.9 ± 0.2 | 166 ± 8 | 9.6 ± 0.2 |
| GRV | 29.3 ± 0.2 | 189 ± 8 | 9.9 ± 0.2 |
| HOP | 28.0 ± 0.3 | 152 ± 10 | 9.0 ± 0.3 |
| LOG | 27.7 ± 0.3 | 130 ± 13 | 8.8 ± 0.2 |
| REE | 29.4 ± 0.2 | 160 ± 9 | 8.7± 0.2 |
| ROA | 29.1 ± 0.2 | 158 ± 8 | 8.3 ± 0.3 |
| RR | 30.1 ± 0.1 | 188 ± 9 | 9.3 ± 0.2 |
| SKN | 29.5 ± 0.2 | 197 ± 12 | 9.3 ± 0.3 |
| SQR | 30.3 ± 0.3 | 223 ± 14 | 9.6 ± 0.2 |
| STB | 29.9 ± 0.1 | 222 ± 9 | 10.3 ± 0.2 |
| TRL | 29.7 ± 0.2 | 197 ± 17 | 9.1 ± 0.3 |
| TT | 30.0 ± 0.2 | 180 ± 6 | 8.9 ± 0.1 |
| XTI | 28.3 ± 0.2 | 169 ± 11 | 8.8 ± 0.2 |

Table S2: Mean tadpole traits (stage, mass, and snout-vent length (SVL)) ± SE across the 14 wood frog populations at the end of the 11-d ranavirus experiment.

| Population | Stage | Mass (mg) | SVL (mm) |
| --- | --- | --- | --- |
| BJ | 36.2 ± 0.3 | 190 ± 20 | 10.4 ± 0.3 |
| BOR | 34.6 ± 0.5 | 156 ± 13 | 10.0. ± 0.3 |
| BOW | 35.9 ± 0.2 | 134 ± 9 | 9.3 ± 0.2 |
| GRV | 37.9 ± 0.3 | 193 ± 11 | 10.1 ± 0.2 |
| HOP | 36.9 ± 0.2 | 168 ± 13 | 9.8 ± 0.2 |
| LOG | 34.8 ± 0.7 | 150 ± 10 | 9.7 ± 0.2 |
| REE | 36.9 ± 0.6 | 191 ± 15 | 9.8 ± 0.3 |
| ROA | 37.8 ± 0.4 | 226 ± 10 | 11.1 ± 0.2 |
| RR | 37.3 ± 0.5 | 211 ± 14 | 10.4 ± 0.3 |
| SKN | 36.5 ± 1.0 | 203 ± 13 | 10.3 ± 0.3 |
| SQR | 36.5 ± 0.4 | 180 ± 11 | 9.8 ± 0.2 |
| TRL | 38.0 ± 0.4 | 222 ± 16 | 10.6 ± 0.2 |
| TT | 37.9 ± 0.7 | 196 ± 15 | 10.1 ± 0.3 |
| XTI | 33.7 ± 0.4 | 156 ± 10 | 9.5 ± 0.2 |

Table S3. Factor loadings, eigenvalues, and percent variance explained derived from the factor analysis for both the trematode and ranavirus experiments.

|  | Trematode experiment | Ranavirus  experiment |
| --- | --- | --- |
| Distance to agriculture | 0.61 | 0.58 |
| Baseline tolerance | 0.82 | 0.84 |
| Plasticity to pesticides | 0.75 | 0.69 |
| Eigenvalue | 2.18 | 2.1 |
| % of variance | 72.6% | 70.1% |

Table S4. Factor scores for both the trematode and ranavirus experiments.

| Population | Trematode experiment | Ranavirus experiment |
| --- | --- | --- |
| BJ | -0.40762 | -0.24711 |
| BOR | 0.21221 | 0.41144 |
| BOW | -0.2708 | -0.20579 |
| GRV | 0.19676 | 0.47855 |
| HOP | -1.37542 | -1.51025 |
| LOG | -0.00922 | 0.09652 |
| REE | -0.80691 | -0.79872 |
| ROA | -0.1759 | 0.03543 |
| RR | 1.1919 | 2.00752 |
| SKN | 0.53209 | 0.81529 |
| SQR | -0.30459 | -0.24275 |
| TRL | 2.41275 | 0.97792 |
| TT | 0.66735 | 0.14124 |
| XTI | -0.12354 | -1.9593 |
| STB | -1.73907 | n/a |

**Figure legends**

Figure S1. Variation in log trematode load across 15 wood frog populations. Values represent means ± 1 SE.

Figure S2. Variation in average tadpole survival (%), time to death (hrs), and log ranavirus load across 14 wood frog populations. Values represent means ± 1 SE.

Figure S3. Relationship between tadpole status (survived or did not survive) 11 d after ranavirus exposure and viral load. Each point represents an individual tadpole.

Figure S4. The number of individual tadpoles surviving the ranavirus experiment by pesticide tolerance mechanism.

Figure S1

Figure S2

Figure S3

Figure S4
