# Supplementary figures and images for: Evolved pesticide tolerance influences susceptibility to parasites in amphibians
Source: Evol Appl. 2017 Jul 4;10(8):802–12. doi: 10.1111/eva.12500 (PMC5680434; doi:10.1111/eva.12500)

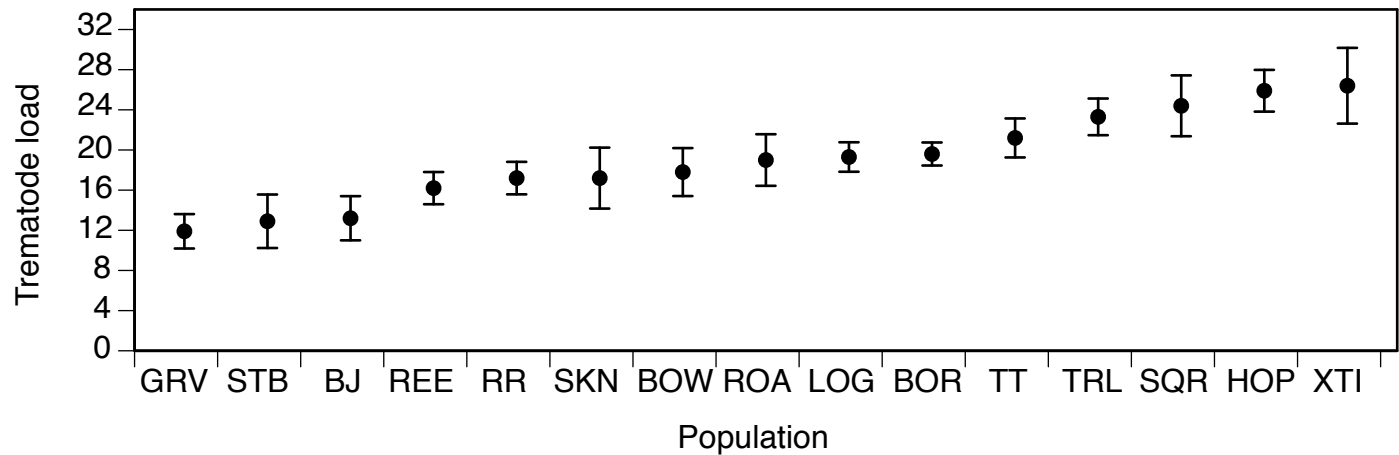

Supplement: Supplementary file 2 [file EVA-10-802-s002.pdf]

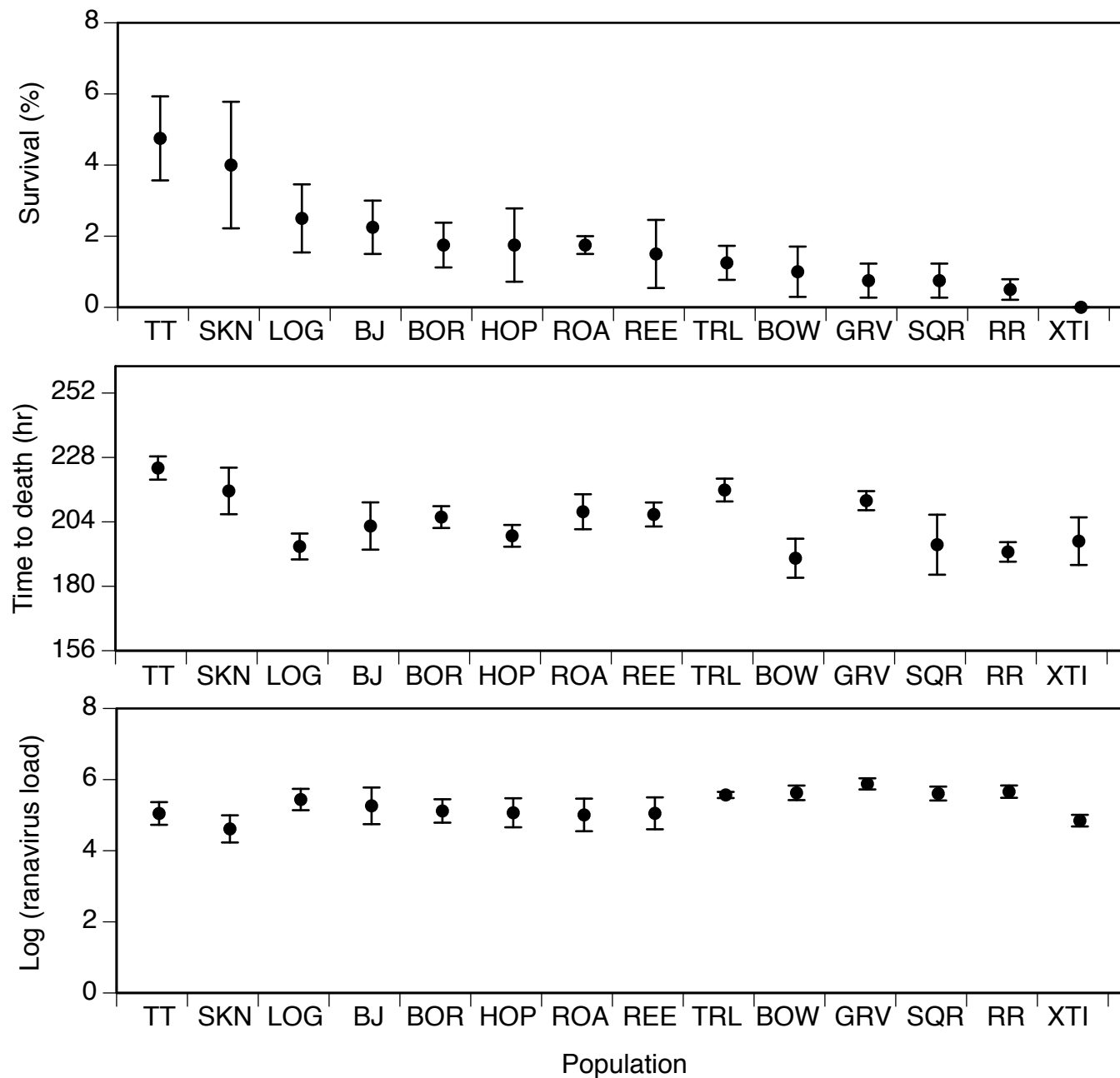

Supplement: Supplementary file 3 [file EVA-10-802-s003.pdf]

# of tadpoles surviving

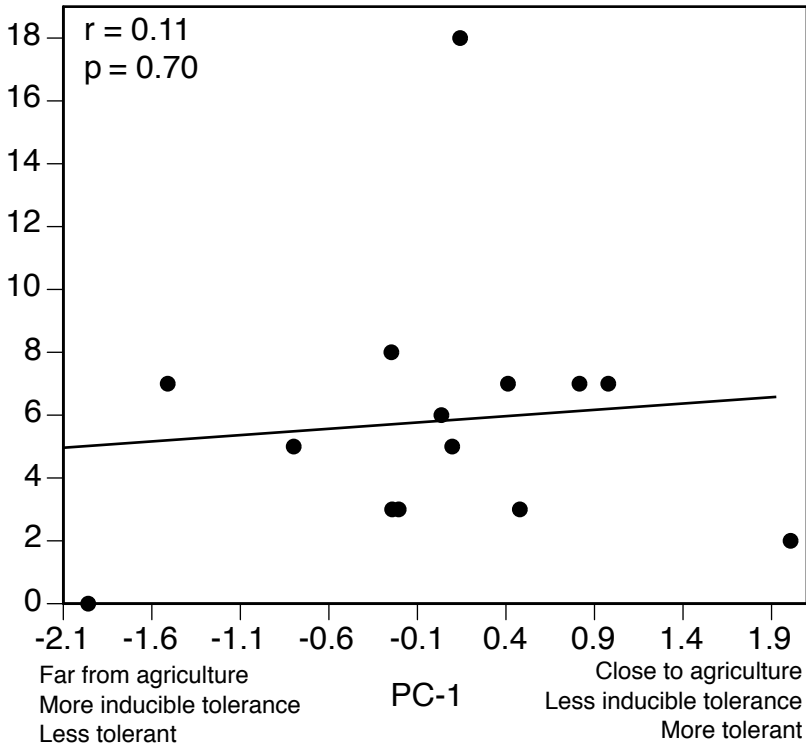

Supplement: Supplementary file 4 [file EVA-10-802-s004.pdf]

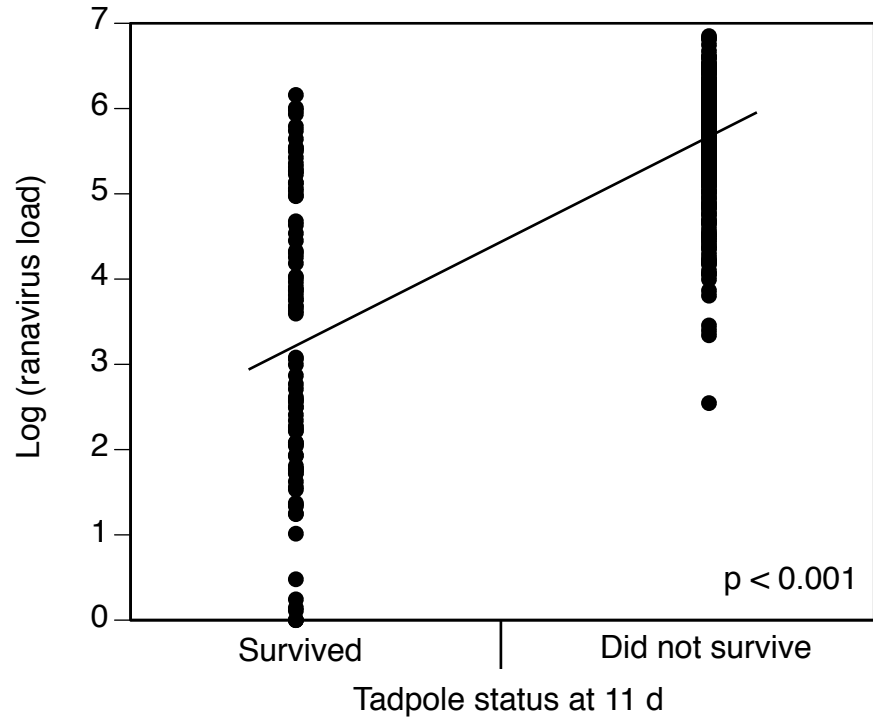

Supplement: Supplementary file 5 [file EVA-10-802-s005.pdf]

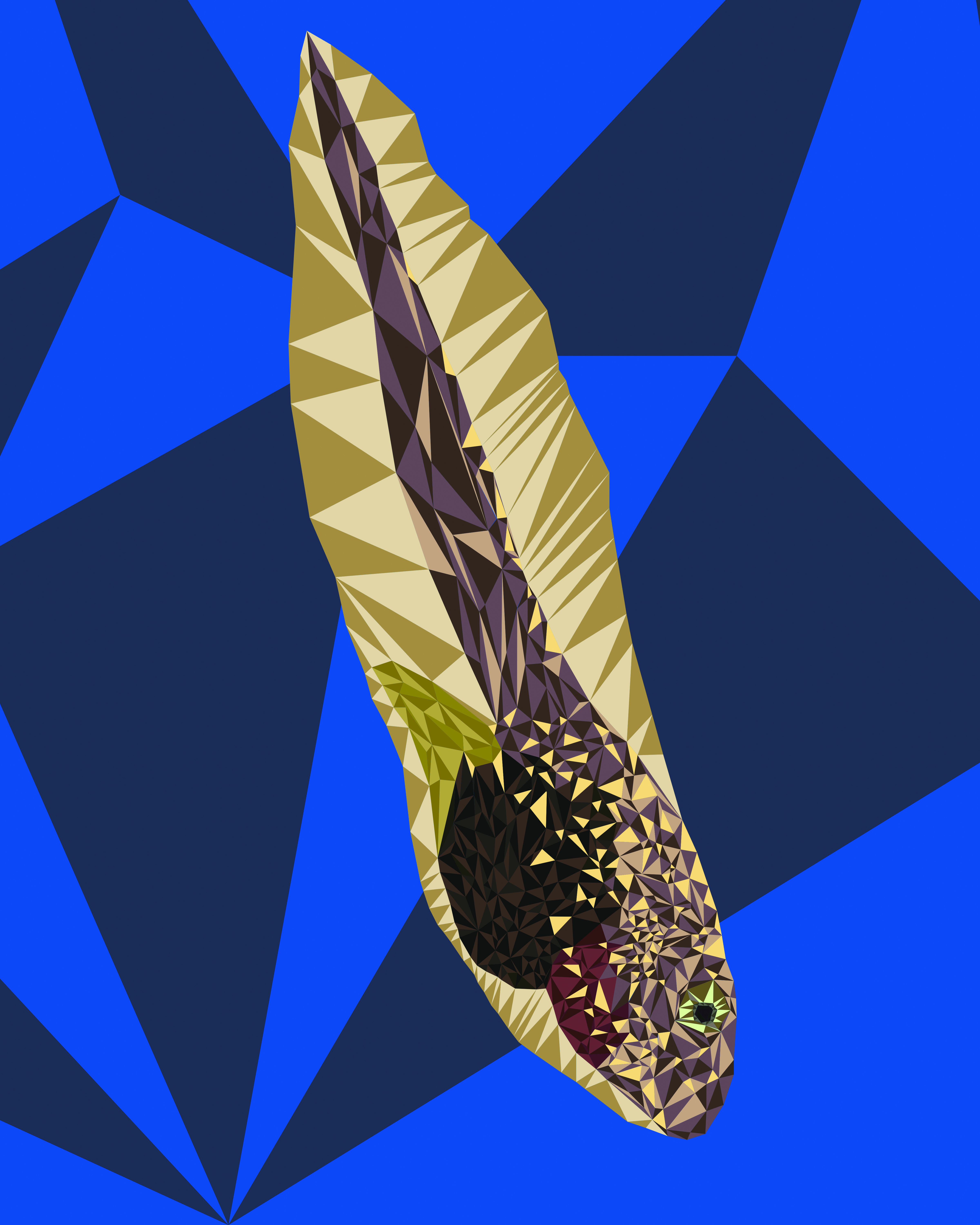

Supplement: Supplementary file 6 [file EVA-10-802-s006.jpg]
