# Supplementary material for: Evolved pesticide tolerance influences susceptibility to parasites in amphibians
Source: Evol Appl. 2017 Jul 4;10(8):802–12. doi: 10.1111/eva.12500 (PMC5680434; doi:10.1111/eva.12500)
Supplement: Supplementary file 7 [file EVA-10-802-s007.docx]

**Appendix-Supplementary tables and figures**

**Tables**

Table A1: Mean tadpole traits (stage, mass, and snout-vent length (SVL)) ± SE across the 15 wood frog populations at the end of the 3-d trematode experiment.

| Population | Stage | Mass (mg) | SVL (mm) |
| --- | --- | --- | --- |
| BJ | 29.8 ± 0.1 | 177 ± 5 | 9.6 ± 0.1 |
| BOR | 28.4 ± 0.3 | 158 ± 8 | 9.5 ± 0.2 |
| BOW | 28.9 ± 0.2 | 166 ± 8 | 9.6 ± 0.2 |
| GRV | 29.3 ± 0.2 | 189 ± 8 | 9.9 ± 0.2 |
| HOP | 28.0 ± 0.3 | 152 ± 10 | 9.0 ± 0.3 |
| LOG | 27.7 ± 0.3 | 130 ± 13 | 8.8 ± 0.2 |
| REE | 29.4 ± 0.2 | 160 ± 9 | 8.7± 0.2 |
| ROA | 29.1 ± 0.2 | 158 ± 8 | 8.3 ± 0.3 |
| RR | 30.1 ± 0.1 | 188 ± 9 | 9.3 ± 0.2 |
| SKN | 29.5 ± 0.2 | 197 ± 12 | 9.3 ± 0.3 |
| SQR | 30.3 ± 0.3 | 223 ± 14 | 9.6 ± 0.2 |
| STB | 29.9 ± 0.1 | 222 ± 9 | 10.3 ± 0.2 |
| TRL | 29.7 ± 0.2 | 197 ± 17 | 9.1 ± 0.3 |
| TT | 30.0 ± 0.2 | 180 ± 6 | 8.9 ± 0.1 |
| XTI | 28.3 ± 0.2 | 169 ± 11 | 8.8 ± 0.2 |

Table A2: Mean tadpole traits (stage, mass, and snout-vent length (SVL)) ± SE across the 14 wood frog populations at the end of the 11-d ranavirus experiment.

| Population | Stage | Mass (mg) | SVL (mm) |
| --- | --- | --- | --- |
| BJ | 36.2 ± 0.3 | 190 ± 20 | 10.4 ± 0.3 |
| BOR | 34.6 ± 0.5 | 156 ± 13 | 10.0. ± 0.3 |
| BOW | 35.9 ± 0.2 | 134 ± 9 | 9.3 ± 0.2 |
| GRV | 37.9 ± 0.3 | 193 ± 11 | 10.1 ± 0.2 |
| HOP | 36.9 ± 0.2 | 168 ± 13 | 9.8 ± 0.2 |
| LOG | 34.8 ± 0.7 | 150 ± 10 | 9.7 ± 0.2 |
| REE | 36.9 ± 0.6 | 191 ± 15 | 9.8 ± 0.3 |
| ROA | 37.8 ± 0.4 | 226 ± 10 | 11.1 ± 0.2 |
| RR | 37.3 ± 0.5 | 211 ± 14 | 10.4 ± 0.3 |
| SKN | 36.5 ± 1.0 | 203 ± 13 | 10.3 ± 0.3 |
| SQR | 36.5 ± 0.4 | 180 ± 11 | 9.8 ± 0.2 |
| TRL | 38.0 ± 0.4 | 222 ± 16 | 10.6 ± 0.2 |
| TT | 37.9 ± 0.7 | 196 ± 15 | 10.1 ± 0.3 |
| XTI | 33.7 ± 0.4 | 156 ± 10 | 9.5 ± 0.2 |

Table A3. Factor loadings, eigenvalues, and percent variance explained derived from the factor analysis for both the trematode and ranavirus experiments.

|  | Trematode experiment | Ranavirus  experiment |
| --- | --- | --- |
| Distance to agriculture | 0.61 | 0.58 |
| Baseline tolerance | 0.82 | 0.84 |
| Plasticity to pesticides | 0.75 | 0.69 |
| Eigenvalue | 2.18 | 2.1 |
| % of variance | 72.6% | 70.1% |

Table A4. Factor scores for both the trematode and ranavirus experiments.

| Population | Trematode experiment | Ranavirus experiment |
| --- | --- | --- |
| BJ | -0.40762 | -0.24711 |
| BOR | 0.21221 | 0.41144 |
| BOW | -0.2708 | -0.20579 |
| GRV | 0.19676 | 0.47855 |
| HOP | -1.37542 | -1.51025 |
| LOG | -0.00922 | 0.09652 |
| REE | -0.80691 | -0.79872 |
| ROA | -0.1759 | 0.03543 |
| RR | 1.1919 | 2.00752 |
| SKN | 0.53209 | 0.81529 |
| SQR | -0.30459 | -0.24275 |
| TRL | 2.41275 | 0.97792 |
| TT | 0.66735 | 0.14124 |
| XTI | -0.12354 | -1.9593 |
| STB | -1.73907 | n/a |

**Figure legends**

Figure A1. Variation in log trematode load across 15 wood frog populations. Values represent means ± 1 SE.

Figure A2. Variation in average tadpole survival (%), time to death (hrs), and log ranavirus load across 14 wood frog populations. Values represent means ± 1 SE.

Figure A3. Relationship between tadpole status (survived or did not survive) 11 d after ranavirus exposure and viral load. Each point represents an individual tadpole.

Figure A4. The number of individual tadpoles surviving the ranavirus experiment by pesticide tolerance mechanism.

Figure A1

Figure A2

Figure A3

Figure A4
